# Description from the Account of the German Army Chaplain Paul De Seur

**Published:** 1919-11-29

**Authors:** 


### EDITH CAVELL'S DEATH.

### Description from the Account of the German Army Chaplain Paul de Seur.

The German Army chaplain, Paul de Seur, attached to the Brussels prison where Edith Cavell was confined, has lately contributed an account of what came under his own observation of her last hours to a German periodical, from which the following has been translated :

"I had asked to be allowed to tell her myself that the sentence was to be carried out next morning, in order to make it a little easier for her. It was very hard for me to carry out my task, but she came to my rescue herself. 'How much time do they give me?' 'Unhappily only until to-morrow morning,' was my answer. For a moment her face flushed and a moisture shone in her eyes, but only for a moment. I offered her my spiritual services and told her I was at her disposal any hour of the day or night. She refused politely, but decidedly. . . .

"I then did something I really had no right to do. I knew and esteemed the Anglican Chaplain, Rev. H. Gahan, who had been allowed to carry on his services (at Christ Church) without any interference. So I asked Miss Cavell if she would like Mr. Gahan to come and administer the Holy Communion to her. Then her eyes lit up, and she accepted with great pleasure. Finally, I told her that it was my duty to be with her during her last minutes. Should I try to arrange that Mr. Gahan should take my place? She refused decidedly. 'It would be much too hard for Mr. Gahan, who was not used to such things,' she said. 'But would you like me to come and fetch you from here, instead of meeting you outside at the Tir National?' (the National Shooting Range). That she accepted thankfully. I said a few words of Christian comfort, and we parted with a warm handshake.

"I hurried quickly to Mr. Gahan's, but he was out. It was already 8.17 P.M. when at last the English clergyman came to me. When I told him confidentially what was to be done he nearly broke down.

"With the permit I had procured for him he went to the prison at St. Gilles. He was allowed to stay with the condemned without witnesses as long as he liked. Later he told me, with the express permission to mention it further, that immediately before her Communion Edith Cavell had said that she saw now, as she stood on the threshold of eternity, that patriotism was not the highest of all things, and that we should hate no one, but love all.

"In the early grey of the morning I set out in the armoured car and drove to the prison. I was announced to Miss Cavell. If I remember rightly, the soldier told me she had just knelt down by her table. A flickering gaslight burned in the cell, and there were two large bunches of withered flowers which had stood there for ten weeks. Miss Cavell had packed her few belongings with the greatest care in a handbag. I conducted her through the long passage of the great prison. The Belgian prison authorities stood there and greeted her silently with the greatest respect. Then we mounted into the armoured car, which was waiting for us in the courtyard. A few moments later the Catholic priest, Father Leyendecker, came out of the same door, with the other condemned prisoner, M. Bancq, an architect of about thirty-five. Bancq went to each one of the German guards standing round, shook hands, and said in Flemish, 'Nicht Nachtragen.'

"And now both cars drove out into the morning. . . .

"As we alighted there was a company under the command of a staff officer standing by. A military judge, with his registrar, an officer of the German Governor, and a doctor were present.

"The company (a firing-party of ten) presented arms. The verdict was read, and we clergy had a last word with the condemned. I grasped Miss Cavell's hand and repeated 2 Cor. xiii. 14. She returned my handshake and answered, 'Tell Mr. Gahan that later on he might say to my dear ones that my life was only lent to me and that I am glad to give it up for my country.' Then I led her the few steps to the stake, to which she was loosely tied. A bandage was tied round her eyes, which the soldier told me were full of tears.

"Then a few seconds passed, which seemed endless to me, because the Catholic priest was talking a little longer to M. Bancq, until he, too, stood by his stake.

"At once a sharp word of command rang out, and two volleys were fired simultaneously by the ten men, at five paces, and without a sound the two victims sank to the ground. A few minutes later the coffins were taken to the graves and lowered, and I prayed over Edith Cavell's grave and said the Lord's Blessing.

"But when I got home I felt sick at heart. I can testify that the whole sad business went off without any accident."

*Reprinted from an article by A. A. B. in the "Record" of November* 20.

